# Sexual health in Tunisian women after menopause: There is a need to improve it to sustain emotional and mental wellbeing

**DOI:** 10.1192/j.eurpsy.2022.2210

**Published:** 2022-09-01

**Authors:** B. Abdelmoula, S. Sellami, I. Bouaziz, E. Khouaja, N. Bouayed Abdelmoula

**Affiliations:** Medical University of Sfax, Genomics Of Signalopathies At The Service Of Medicine, Sfax, Tunisia

**Keywords:** Sexual health, menopause

## Abstract

**Introduction:**

Maintaining sexuality is important to the well-being of women, particularly after menopause and benefits of sexual satisfaction in terms of emotional well-being and quality of life have been well demonstrated.

**Objectives:**

This study aims to assess the sexual health behaviors in Tunisian women during and after menopause and the awareness of Tunisian partners about the role of the quality of their sexuality regarding their physical and psychological wellbeing.

**Methods:**

We comprehensively review the scientific literature using Pubmed database to state Tunisian literature regarding sexual behaviors and function in women during and after menopause. Interviews with twenty Tunisian women after menopause about sexual health have been conducted.

**Results:**

Our bibliographic research revealed a poor literature with only two papers responding to our inquiry but among a specific female population investigated after experiencing breast cancer “Female sexuality in premenopausal patients with breast cancer on endocrine therapy and sexuality after breast cancer: cultural specificities of Tunisian population”. Interrogated women reported a poor sexual satisfaction as well as sexual difficulties in the partner or with him. In fact, there is an important wrong understanding of the female anatomy and physiology by both partners, for the female sexual satisfaction. There is also many wrong cultural ideas about menopause and sexuality.

**Conclusions:**

Currently, sexuality in Tunisian women during and after menopause is influenced by ageing, by previous sexual function and experiences, the male domination in partner’s sexual practices and the sexual functioning in the partner. In general, there is an unfavorable body image and disturbed sexual health.

**Disclosure:**

No significant relationships.

